# The regulation of auxin receptor gene *CsAFB2* by csn‐miR393a confers resistance against *Colletotrichum gloeosporioides* in tea plants

**DOI:** 10.1111/mpp.13499

**Published:** 2025-03-28

**Authors:** Anburaj Jeyaraj, Shujing Liu, Rui Han, Yuxin Zhao, Tamilselvi Elango, Yuhua Wang, Xuan Chen, Jing Zhuang, Xinghui Li

**Affiliations:** ^1^ College of Horticulture Nanjing Agricultural University Nanjing China

**Keywords:** anthracnose disease resistance, *Camellia sinensis*, *Colletotrichum gloeosporioides*, host–pathogen interaction, microRNAs, target mimicry

## Abstract

Anthracnose, a severe disease caused by *Colletotrichum*, affects diverse crops and leads to significant economic losses through pronounced fruit/leaf lesions. MicroRNAs (miRNAs) play crucial roles in modulating gene expression in response to disease resistance, defence responses and plant immunity. However, the regulatory mechanisms of miRNAs in responses to *Colletotrichum gloeosporioides* remain unknown in tea plants. Our study revealed that csn‐miR393a targets auxin receptor gene *CsAFB2* during resistance to *C. gloeosporioides* in tea plants by comparing the resistant cultivar Zhongcha108 to the susceptible cultivar Longjing43. Through *Nicotiana benthamiana* leaf co‐transformation assays, we demonstrated that csn‐miR393a suppresses the expression of *CsAFB2*, and csn‐miR393a target mimic blocks the function of csn‐miR393a, leading to increase in the expression of *CsAFB2*. Repression of transcripts in tea leaves by antisense oligonucleotides demonstrated that csn‐miR393a negatively affects the tea plant defence by regulating reactive oxygen species homoeostasis, *PR* gene expression and catechin accumulation. To further validate the regulatory mechanisms of csn‐miR393a, we developed transgenic tea plants overexpressing *CsAFB2*, resulting in enhanced resistance responses against *C. gloeosporioides*. Additionally, transgenic *N. benthamiana* lines overexpressing a csn‐miR393a target mimic provided further evidence that csn‐miR393a negatively regulates the tea plant defence response against *C. gloeosporioides* by suppressing *CsAFB2*. Therefore, manipulating csn‐miR393a or its target gene, *CsAFB2*, has the potential to strengthen the tea plant's resistance against tea anthracnose.

## Graphical abstract

Overexpression of csn‐miR393a target mimicry blocks the function of csn‐miR393a, thereby increasing the expression of *CsAFB2*, which positively regulates auxin signalling to enhance plant resistance.

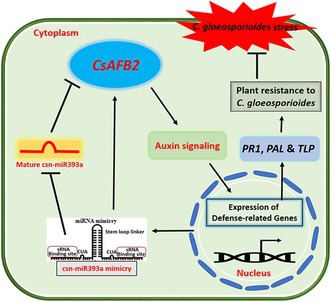

## 1 INTRODUCTION

The economically important plantation crop known as tea plant (*Camellia sinensis*) is mainly grown in tropical and subtropical regions. Numerous biotic stressors restrict the growth and development of tea plants (Mukhopadhyay et al., 2016). Among these, anthracnose caused by *Colletotrichum gloeosporioides* is one of the most destructive foliar diseases that affect tea plants, which results in significant losses of crop and tea production worldwide (Jeyaraj et al., 2019). To penetrate and colonize host plants, *C. gloeosporioides* uses a hemibiotrophic mode of infection, in which biotrophic and necrotrophic phases occur successively (Münch et al., 2008). *C. gloeosporioides* can cause severe damage that eventually results in defoliation, which affects both young and old leaves (Fang et al., 2013). It was previously reported in Anhui province that 30%–60% of the tea fields showed signs of brown blight on leaves of anthracnose‐infected plants, and it also caused 30%–50% decreased tea yield in Guangdong province of China (Guo et al., 2014; Shi et al., 2018). Consequently, the development of plants resistant to pathogen has become a major focus for anthracnose disease prevention and management.

Generally, plants have a strong innate immune system for protection against pathogen invasion. In response to biotic stressors, plants activate two defence mechanisms against pathogens: pathogen‐associated molecular pattern (PAMP)‐triggered immunity (PTI) and effector‐triggered immunity (ETI) (Jones & Dangl, 2006). PTI, the first line of active defence, is rapidly triggered the host's identification of extracellular PAMPs by surface pattern recognition receptors (PRRs). To overcome the basal defence, pathogens have evolved mechanisms to distribute pathogen virulence factors (effectors) to suppress PTI. At this stage, disease‐resistance (R) proteins in resistant plants recognize these effectors within the cells to activate the ETI mechanism (Pruitt et al., 2021). Previously, several studies have shown that microRNAs (miRNAs) are involved in the PTI and ETI defence mechanisms during plant fungal infection (Huang et al., 2016; Weiberg et al., 2014). Plant miRNAs are a class of small endogenous non‐coding RNA molecules that interact with their target mRNAs and negatively regulate post‐transcriptional gene expression (Jones‐Rhoades et al., 2006). miRNAs have been reported to play crucial roles in the regulation of diverse cellular pathways and participate in most biological processes, including plant development, hormone signalling and responses to abiotic/biotic stresses (Chen, 2009; Sunkar, 2010). For example, poplar miR472a plays a key role in plant immunity to *C. gloeosporioides* and *Cytospora chrysosperma* by targeting NBS‐LRR transcripts (Su et al., 2018). The manipulation of target gene expression by overexpression of miRNAs or their short tandem target mimic (STTM) can be used as a new strategy to explore the activities of miRNAs to enhance plant disease resistance (Su et al., 2018). In *Arabidopsis*, overexpression of miR858 increases the plant's vulnerability to *C. higginsianum* infection, whereas inhibition of miR858 activity by target mimics increases the plant's resistance to *C. higginsianum* (Camargo‐Ramírez et al., 2018).

miR393, a highly conserved miRNA family in plants, is involved in both biotic and abiotic stress responses (Guo et al., 2016; Jiang et al., 2022). miR393 had been studied in a number of species, including *Arabidopsis* (Navarro et al., 2006), poplar (Lu et al., 2008), rice (Bian et al., 2012) and barley (Yuan et al., 2019), and it was found that the miR393 family regulates auxin signalling pathway via down‐regulation of auxin receptor genes encoding transport inhibitor response 1 (TIR1) and auxin signalling F‐box (AFBs) proteins (Jiang et al., 2022). The miRNA‐mediated regulation of target genes in auxin signalling mechanism might be related to plant defence responses (Yin et al., 2013). Overexpression of miR393 represses auxin signalling, increasing susceptibility to necrotrophic pathogens and resistance to biotrophic pathogens (Robert‐Seilaniantz et al., 2011). In rice, overexpression of miR393 increases plant susceptibility to the rice black‐streaked dwarf virus (RBSDV) (Zhang et al., 2019). In cotton, ghr‐miR393 knockdown increased susceptibility to *Verticillium dahliae* infection, but overexpression of ghr‐miR393 strengthened plant resistance to the infection (Shi et al., 2022). However, miR393‐mediated defence against the hemibiotrophic pathogen *C. gloeosporioides* remains unknown in tea plant. In our previous study, we observed differential expression of miR393a in the leaves of susceptible cultivar Longjing43 (LJ43) and resistant cultivar Zhongcha108 (ZC108) in response to *C. gloeosporioides* stress (Jeyaraj et al., 2021). To investigate the role of csn‐miR393a in the regulation of plant immune responses against *C. gloeosporioides* infection, we analysed the expression pattern of csn‐miR393a and its target gene, *CsAFB2*, in the leaves of LJ43 and ZC108 after *C. gloeosporioides* infection. We demonstrated csn‐miR393a negatively regulated tea plant defence against *C. gloeosporioides* by an antisense oligonucleotide (asODN) assay. We generated *CsAFB2*‐transformed lines through *Agrobacterium rhizogenes*‐mediated tea root transformation to reveal its role in the resistance to *C. gloeosporioides*. Additionally, we constructed *Nicotiana benthamiana* transgenic lines overexpressing csn‐miR393a (OX‐R393a) and its target mimic (MIM‐R393a) for an in‐depth study to elucidate the role of csn‐miR393a in plant resistance against *C. gloeosporioides* infection. In summary, our data demonstrated that overexpression of *CsAFB2* in transformed tea lines and overexpression of a csn‐miR393a target mimic in *N. benthamiana* resulted in enhanced resistance to *C. gloeosporioides* infection.

## 2 RESULTS

### 2.1 csn‐miR393a and its target gene *CsAFB2* are related to *C. gloeosporioides* resistance

**FIGURE 1:**
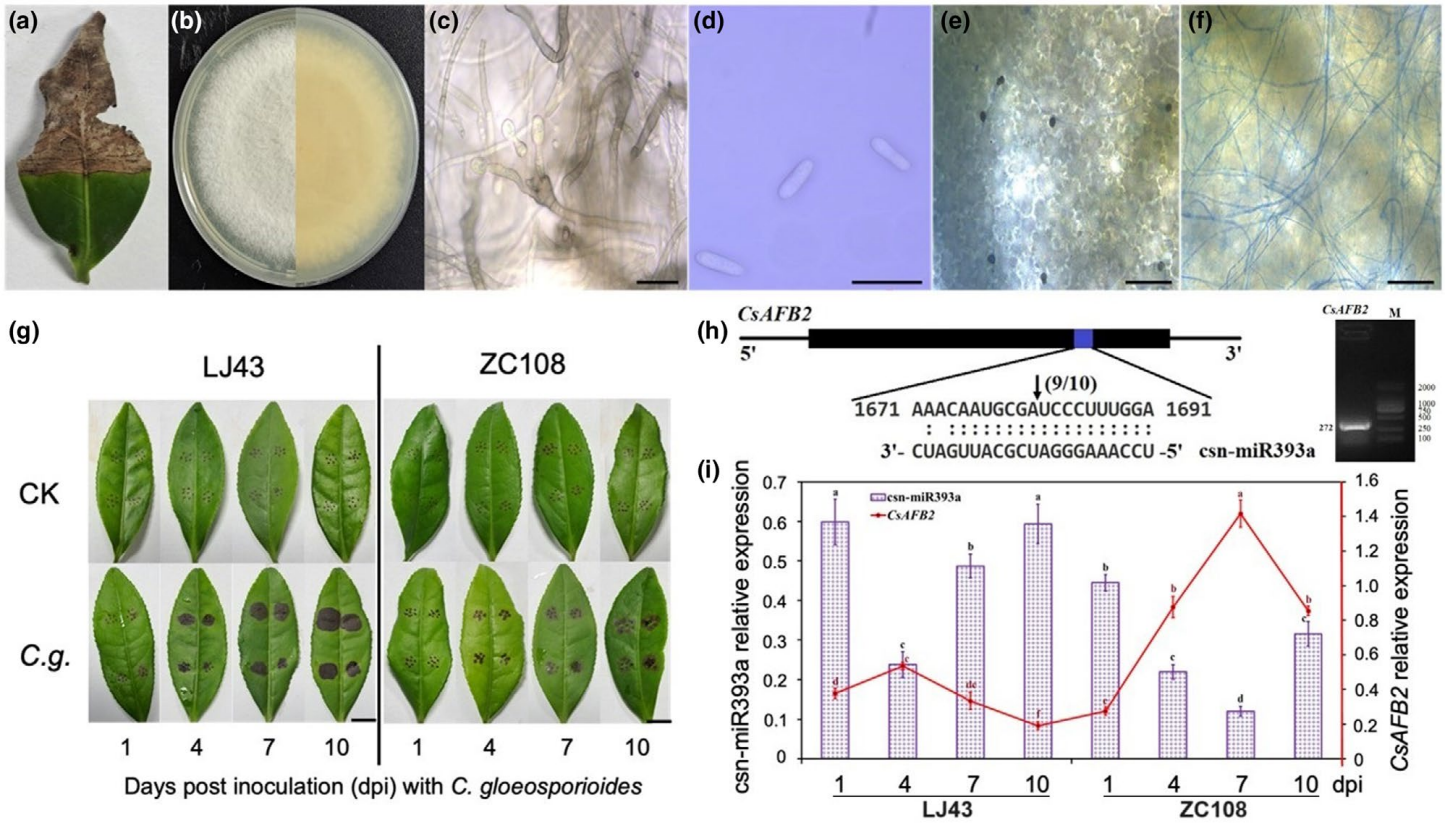
csn‐miR393a and its target gene *CsAFB2* are related to *Colletotrichum gloeosporioides* resistance. (a–f) Morphological characteristics of *C. gloeosporioides*: symptom on tea leaf (a), forward and reverse view of 6‐day‐old potato dextrose agar culture (b), conidiophores (c), conidia (d), infection structure (appressorium) of *C. gloeosporioides* at 24 h (e) and filamentous mycelial development at 48 h (f). Bars (c,d) = 20 μm and bars (e,f) = 50 μm. (g) Pathogenicity of *C. gloeosporioides* on two cultivars of tea plant leaves (LJ43 and ZC108) at 1, 4, 7 and 10 days post‐inoculation (dpi): *C.g*. represents *C. gloeosporioides*‐inoculated leaves, and CK represents mock‐inoculated control. Bars = 1 cm. (h) Experimental validation of csn‐miR393a targets using 5′ RLM‐RACE, and agarose gel electrophoresis of csn‐miR393a guided cleavage product. The upper rectangular boxes represent the gene structure of *CsAFB2*. Top strand depicts the csn‐miR393a complementary site, and bottom strand depicts the anti‐parallel csn‐miR393a. The arrows indicate the cleavage site verified by RLM‐RACE products with the frequency of clones shown above. (i) The expression analysis of csn‐miR393a and its target gene, *CsAFB2*, compared to the mock‐inoculated (CK) control. The bars and lines represent the means and *SD* values of three biological replicates, respectively, in tea leaves exposed to *C. gloeosporioides* at different time intervals by reverse transcription‐quantitative PCR. Different letters above the bars represent significant differences at α = 0.05 by Duncan's mutiple‐range test.

We obtained *C. gloeosporioides* cultures from infected tea leaves using the single‐spore isolation method (Figure 1a). The cultures of *C. gloeosporioides* on potato dextrose agar (PDA) were white and grey on the front side, with white and dark grey on the back side of the colonies (Figure 1b). Conidiophores, conidia and colonization of *C. gloeosporioides* in the host tissue were examined for microscopic morphology (Figure 1c–f). Based on the rDNA internal transcribed spacer (ITS) sequencing analysis, the fungus was confirmed as *C. gloeosporioides* (JX010223.1). To investigate the pathogenicity of *C. gloeosporioides*, we used a conidial suspension (2 × 10^5^ spores/mL) to inoculate the leaves of LJ43 and ZC108 and, then, monitored disease symptoms at 1, 4, 7 and 10 days post‐inoculation (dpi) (Figure 1g). At 1 dpi, circular and elliptical brown spots appeared on the leaves of LJ43. At 7–10 dpi, the leaves of LJ43 showed a gradual progression of the disease development, with leaves being almost completely covered by circular brown sunken spots. In contrast, the leaves of ZC108 exhibited tiny brown spots around wounded areas at 4 dpi, enlarging to circular brown sunken spots at 7 and 10 dpi. The difference in disease severity between LJ43 and ZC108 showed that ZC108 exhibited higher resistance to *C. gloeosporioides* infection compared to LJ43. There were no symptoms in the control leaves during the incubation period (Figure 1g). Our previous small RNA sequencing data revealed differential responses of csn‐miR393a to *C. gloeosporioides* infection in the susceptible (LJ43) and resistant (ZC108) cultivars (Jeyaraj et al., 2021). *CsAFB2* was predicted to be the target of csn‐miR393a during *C. gloeosporioides* infection (Jeyaraj et al., 2021) and we proposed the regulatory module, csn‐miR393a‐*CsAFB2* may participate in the resistance of tea plants to *C. gloeosporioides* infection. To identify the cleavage site of the csn‐miR393a target, we conducted RNA ligase‐mediated rapid amplification of 5′ cDNA ends (5′ RLM‐RACE) analysis, confirming that the cleavage site of *CsAFB2* lies between the 10th and 11th bases from the 5′ end pairing of csn‐miR393a (Figure 1h). To investigate the expression patterns of csn‐miR393a and its target gene (*CsAFB2*) in the susceptible and resistant tea cultivars under *C. gloeosporioides* stress, we conducted reverse transcription‐quantitative PCR (RT‐qPCR) assay. The results showed that in the leaves of susceptible cultivar LJ43, csn‐miR393a had 0.60‐, 0.24‐, 0.49‐ and 0.59‐fold expression levels at 1, 4, 7 and 10 dpi, respectively, when compared to the mock‐inoculated control (CK). In the resistant tea cultivar ZC108, the expression levels of csn‐miR393a were 0.44‐, 0.22‐, 0.12‐ and 0.32‐fold at 1, 4, 7 and 10 dpi, respectively, compared to the CK control. These findings indicated that the expression level of csn‐miR393a displayed significant up‐regulation after exposure to *C. gloeosporioides* in LJ43, except at 4 dpi, possibly representing early classical defence responses against *C. gloeosporioides* during its biotrophic phase; however, at 7 and 10 dpi, the expression level of csn‐miR393a progressively increased, which might be associated with the necrotrophic phase in the susceptible LJ43. In the resistant cultivar ZC108, csn‐miR393a expression was down‐regulated at 4–10 dpi compared to LJ43 (Figure 1i). Understanding the regulatory networks mediated by miRNA in response to *C. gloeosporioides* stress depends on the expression analysis of the target gene. The *CsAFB2* expression was inversely correlated with csn‐miR393a (Figure 1i). In the resistant cultivar ZC108, csn‐miR393a was down‐regulated in response to *C. gloeosporioides* infection, suggesting a specific role during the tea plant–*C. gloeosporioides* interaction.

Anthracnose, caused by *C. gloeosporioides*, affects a variety of plants. To explore the conservative function of csn‐miR393a, we conducted a phylogenetic analysis of csn‐miR393a among diverse plant species, demonstrating the evolutionary relationships of csn‐miR393a (Figure S1a). Additionally, we studied the phylogeny of AFB2 using 12 distinct protein sequences from different plant species, constructing a neighbour‐joining phylogenetic tree. Our findings revealed that CsAFB2 shared the highest similarity with the AFB2 proteins of *Diospyros lotus* compared to other plant species (Figure S1b). Conserved domain analysis indicated that CsAFB2 contains F‐box/TIR domains. According to Compute pI/Mw analysis, the deduced protein sequences of CsAFB2 has a theoretical molecular weight of 70.83 kDa, with an isoelectric point of 7.71. Collectively, these analyses suggest that csn‐miR393a‐mediated *CsAFB2* regulation could be conserved in different plant species.

### 2.2 csn‐miR393a regulates *CsAFB2* expression

**FIGURE 2:**
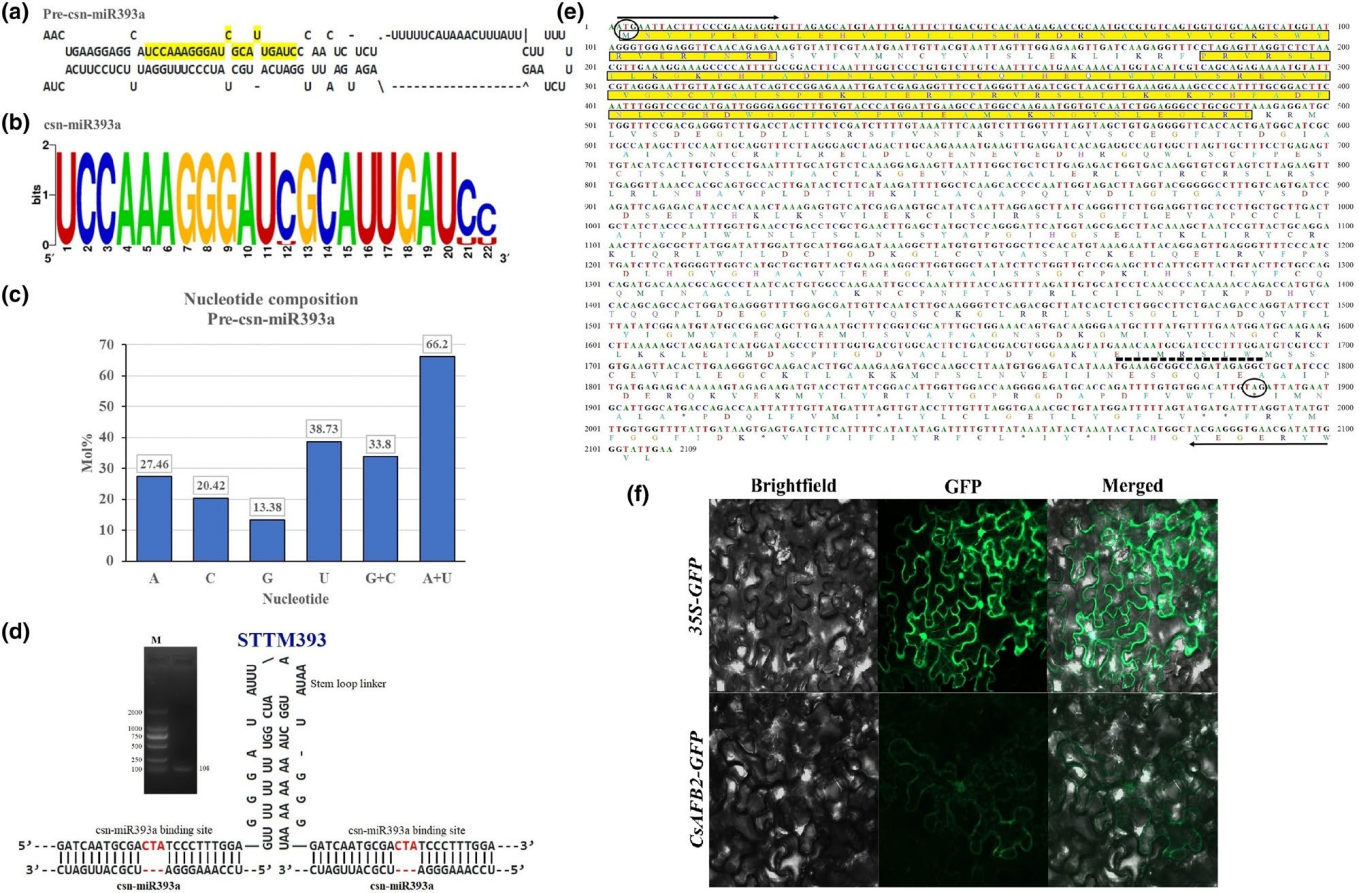
Analysis of mature/precursor csn‐miR393a, csn‐miR393a target gene (*CsAFB2*) and target mimic sequences. (a) Folded hairpin structure of csn‐MIR393a in *Camellia sinensis*. (b) Sequence logo of mature csn‐miR393a sequences. (c) Nucleotide composition in the csn‐MIR393a. (d) Structure of csn‐miR393a specific target mimic designed to suppress miRNA expression. (e) Complete cDNA and derived amino acid sequences of *CsAFB2*. Black underlined sequences indicate gene‐specific primers for reverse transcription‐PCR. Arrows indicate direction of the primer. Triple bases in the circle show the start and stop codons. Sequences underlined with dotted line showed the cleavage site of csn‐miR393a. (f) Subcellular localization assay of *CsAFB2* in epidermal cells of *Nicotiana benthamiana* leaves.

**FIGURE 3:**
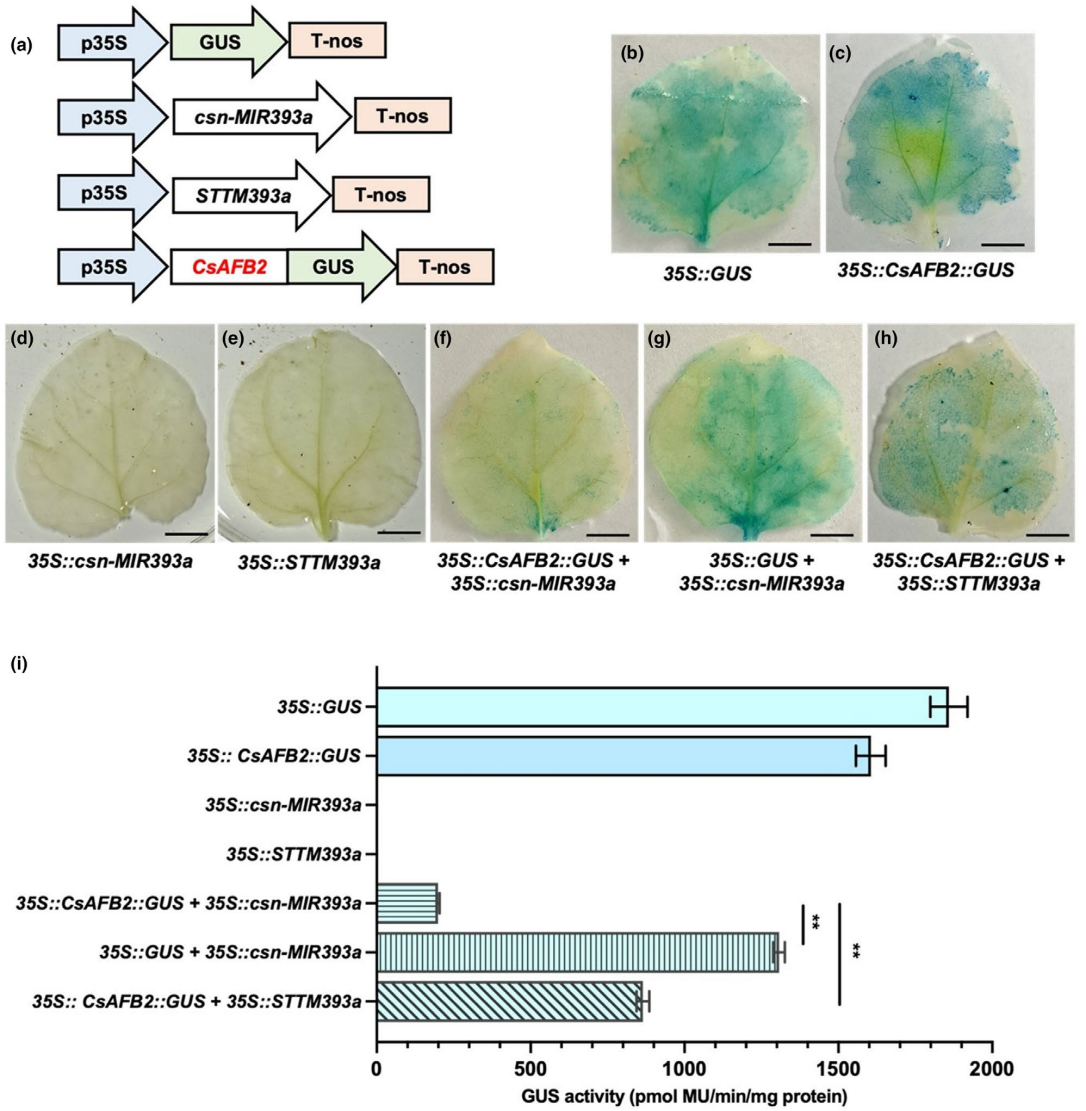
csn‐miR393a represses *CsAFB2* expression. (a) The design of constructs. (b–h) The *35S::GUS* (b), *35S::CsAFB2:GUS* (c), *35S::csn‐miR393a* (d), *35S::STTM393a* (e), *35S::CsAFB2:GUS* combined with *35S::csn‐miR393a* (f), *35S::GUS* combined with *35S::csn‐miR393a* (g) and *35S::CsAFB2:GUS* combined with *35S::STTM393a* (h) constructs were transformed into *Nicotiana benthamiana* leaves using *Agrobacterium*‐mediated infiltration. β‐glucuronidase (GUS) phenotype was observed by histochemical staining. Bars = 1 cm. (i) Quantitative detection of GUS activity in the leaves inoculated with different fusion constructs shown in (b–h). **p < 0.01 (*t* test).

The precursor of csn‐miR393a was isolated from tea plant genomic DNA and sequenced. The results indicated that the length of precursor csn‐miR393a (csn‐MIR393a) was 142 bp, with A + U content of 66.2% (Figure 2a–c). To investigate the subcellular localization of the CsAFB2 protein, the open reading frame (ORF) of *CsAFB2* was amplified (Figure 2e) and used to generate a *35S::CsAFB2*‐*GFP* construct. This recombinant construct was transiently transformed into the lower epidermis of *N. benthamiana* leaves. The results showed that, compared with the control, the green fluorescence signals of CsAFB2 were preferentially detected in the plasma membrane and cytoplasm of the leaf epidermis (Figure 2f). To test the cleavage function of csn‐miR393a on its predicted target *CsAFB2*, a co‐transformation experiment was performed in *N. benthamiana* leaves using the vector pBI121 containing the *gusA* reporter gene driven by the CaMV 35S promoter (Figure 3a). The vector pBI121 carrying *35S::GUS*) served as the positive control. The β‐glucuronidase (GUS) phenotype was observed via histochemical staining of transiently transformed leaves. To reduce repression efficiency of csn‐miR393a to *CsAFB2*, we designed the artificial target mimic for csn‐miR393a (STTM393). STTM393 contained two copies of the mimic target site (24 nucleotides) with a 48‐nucleotide linker (Figure 2d). The leaves inoculated with *35S::GUS* and *35S::CsAFB2*::*GUS* showed GUS signals, whereas no GUS phenotype was observed in the leaves inoculated with *35S::csn‐MIR393a* or *35S::STTM393* (Figure 3b–e). Interestingly, GUS expression was significantly lower in the leaves co‐transformed with *35S::CsAFB2::GUS* and *35S::csn‐MIR393a* compared to the leaves inoculated with *35S*::*GUS* and *35S::csn‐MIR393a* (Figure 3f,g). When *35S::CsAFB2::GUS* was co‐transformed along with *35S::STTM393a*, the expression of GUS was increased compared with *35S::CsAFB2:GUS* and *35S::csn‐MIR393a* co‐transformed leaves (Figure 3f,h). To confirm the results of the histochemical observations, GUS activity was assayed quantitatively in the leaves inoculated with different recombinant vectors; the results were consistent with the histochemical observations (Figure 3i). The reduced GUS signal in the leaves with *35::GUS* and *35S::csn‐miR393a* compared to the single inoculation with *35::GUS* could be attributed to variations in bacterial suspension. One millilitre of bacterial suspension was injected into leaves for the single construct, whereas two 0.5 mL volumes with each construct were used for the combined treatment. Altogether, these findings indicate that csn‐miR393a targets and suppresses the expression of *CsAFB2*, and a target mimic of csn‐miR393a reduces the function of csn‐miR393a in *N. benthamiana* leaves.

### 2.3 csn‐miR393a‐mediated *CsAFB2* expression is involved in the tea plant defence response against *C. gloeosporioides*

**FIGURE 4:**
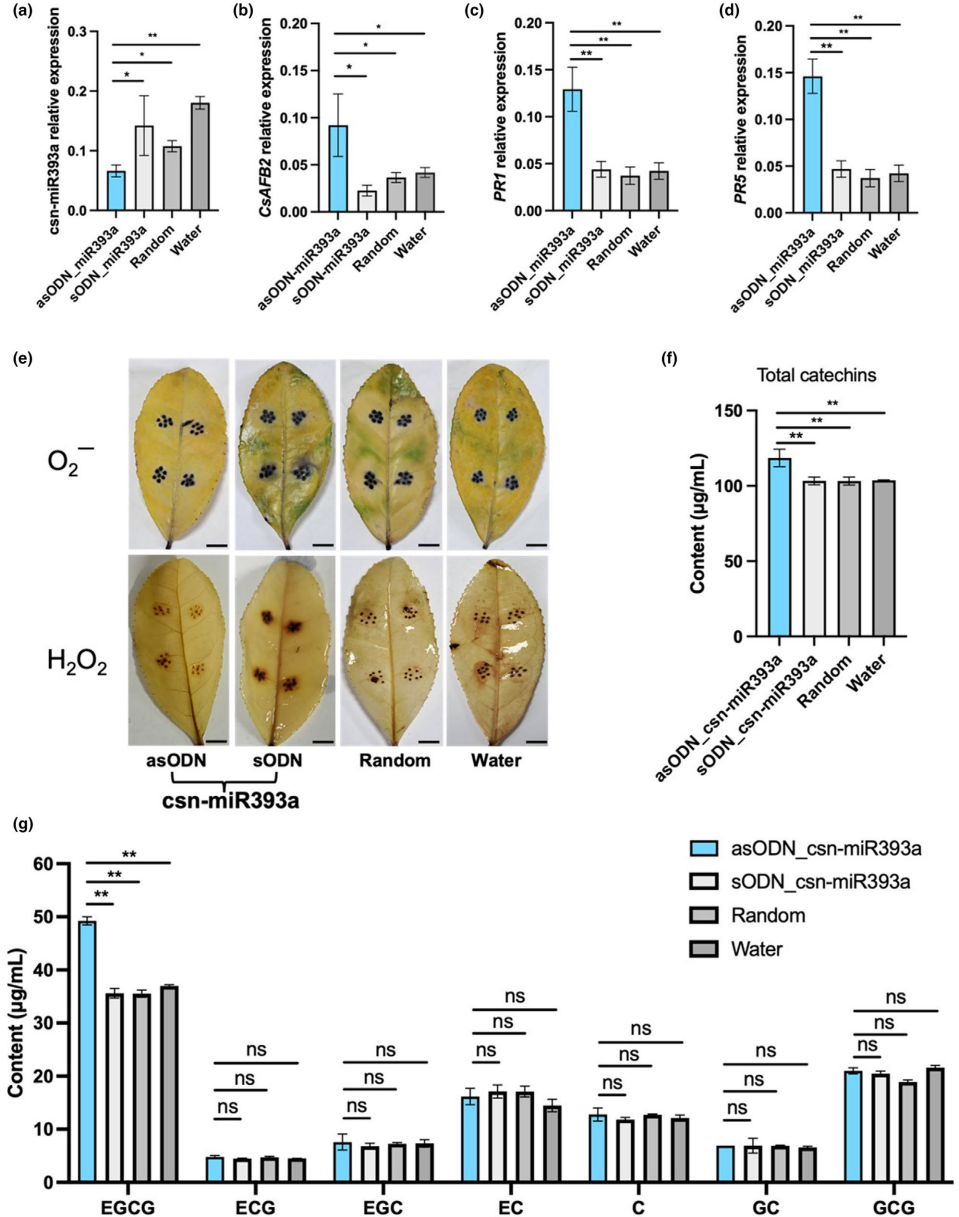
csn‐miR393a‐mediated *CsAFB2* expression is involved in the tea plant defence response against *Colletotrichum gloeosporioides* as shown by an antisense oligonucleotide (asODN) assay. (a–d) The expression levels of csn‐miR393a (a), *CsAFB2* (b), *PR1* (c) and *PR5* (d) in the controls and asODN‐treated tea leaves. (e) Nitroblue tetrazolium (NBT) and 3,3′‐diaminobenzidine (DAB) staining of $O_{2}^{-}$ and H_2_O_2_ in the controls and asODN‐treated tea leaves 48 h after *C. gloeosporioides* inoculation. Bars = 0.5 cm. (f) Contents of catechins in the controls and asODN‐treated tea leaves. (g) Contents of epigallocatechin gallate (EGCG), epicatechin gallate (ECG), epigallocatechin (EGC), epicatechin (EC), catechin (C), gallocatechin (GC) and gallocatechin gallate (GCG) in the controls and asODN‐treated tea leaves. ns indicates not significant, *p < 0.05, **p < 0.01 (*t* test).

To further investigate the function of csn‐miR393a in response to *C. gloeosporioides* in tea plants, we knocked down csn‐miR393a in tea leaves using a transcript‐specific antisense oligonucleotide (asODN) to monitor the expression level of csn‐miR393a. The control leaves were incubated with water, random oligonucleotides and sense oligonucleotides (sODNs). The RT‐qPCR analysis showed that tea leaves incubated with asODNs had considerably lower transcript levels of csn‐miR393a than control leaves incubated with sODNs, random oligonucleotides or water (Figure 4a). Knockdown of the transcript levels of csn‐miR393a led to increased *CsAFB2* expression in tea leaves after incubation (Figure 4b). These findings affirmed the efficacy of the asODN technique and its applicability to manipulate miRNA in tea plants. The expression analysis of pathogenesis‐related genes *PR1* and *PR5* revealed that, compared to the controls, the expression levels of *PR1* and *PR5* were up‐regulated in the asODN‐csn‐miR393a‐treated tea leaves (Figure 4c,d).

The production of reactive oxygen species (ROS) is a typical response to pathogen infection (Mittler et al., 2022). To determine whether suppression of csn‐miR393a affected the plant defence responses to *C. gloeosporioides* infection, in situ localization of the ROS hydrogen peroxide (H_2_O_2_) and superoxide radical ($O_{2}^{-}$) was examined by 3,3′‐diaminobenzidine (DAB) and nitroblue tetrazolium (NBT) staining, respectively. The results showed that, compared to sODN‐treated tea leaves, the accumulation of $O_{2}^{-}$ and H_2_O_2_ was decreased in the asODN‐csn‐miR393a‐treated tea leaves (Figure 4e). Catechins are natural antifungal compounds (Jiang et al., 2015; Wang, Qian, et al., 2016). The total amount of catechins was higher in the asODN‐csn‐miR393a‐treated tea leaves than in other controls (Figure 4f). Specifically, the epigallocatechin gallate (EGCG) contributed to this total catechin accumulation, whereas epicatechin gallate (ECG), epigallocatechin (EGC), epicatechin (EC), catechin (C), gallocatechin (GC) and gallocatechin gallate (GCG) in the sODNs‐csn‐miR393a‐treated tea leaves were not significantly changed (Figure 4g). Altogether, these observations indicated that suppression of csn‐miR393a could increase the resistance of tea plants to *C. gloeosporioides* infection.

### 2.4 Overexpression of *CsAFB2* in tea root enhances the resistance of tea plant to *C. gloeosporioides*

**FIGURE 5:**
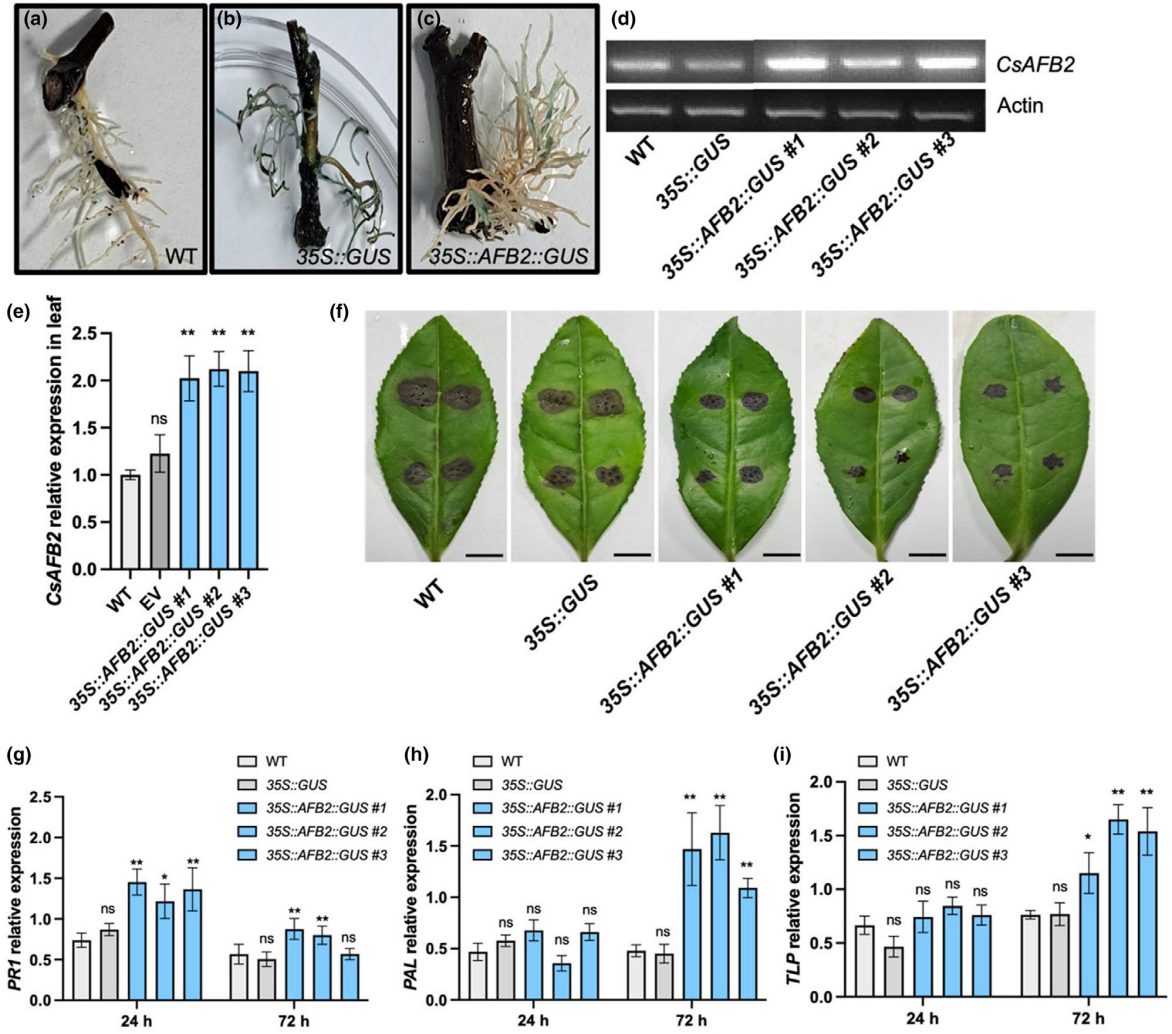
*CsAFB2‐*overexpressing transgenic tea plants show resistance against *Colletotrichum gloeosporioides*. (a–c) β‐glucuronidase (GUS) histochemical staining of the control (wild type, WT) (a) and transformed roots of *Camellia sinensis* (b,c). *35S::GUS*, transgenic roots carrying the empty vector (pBI121); *35S::CsAFB2:GUS*, transgenic roots overexpressing *CsAFB2*. (d) The *CsAFB2* expression in the roots of controls and transformed tea lines by semiquantitative PCR. (e) The *CsAFB2* expression in the leaves of controls and transformed tea lines by reverse transcription‐quantitative PCR. (f) Pathogenicity of *C. gloeosporioides* on the leaves of *CsAFB2*‐overexpressing transgenic lines at 5 days post‐inoculation. Bars = 1 cm. (g–i) The expression levels of *PR1* (g), *PAL* (h) and *TLP* (i) genes in the leaves of controls (wild‐type, WT; empty vector, *35S::GUS*) and *C. gloeosporioides*‐inoculated transformed tea lines. ns, not significant, *p < 0.05, **p < 0.01 (*t* test) compared to WT.

To examine the functional role of the csn‐miR393a*‐CsAFB2* module, we generated transgenic tea plants overexpressing *CsAFB2* using *Agrobacterium rhizogenes*‐mediated root transformation in tea cuttings (Figure S2). The recombinant plasmid *35S::CsAFB2‐GUS* was transformed into the root system of LJ43. Tea roots infected with wild‐type *A. rhizogenes* (WT) and transformed with the pBI121 vector carrying *35S::GUS* were used as controls. A total of 20 independent tea plants were used for each root transformation. To confirm the integration of the T‐DNA region in the transformed roots, we isolated genomic DNA from all the plants and tested for the *gusA* fragments (1635 bp) using PCR amplification. Among the 20 plants, six and nine transformants of *35S::GUS* and *35S::CsAFB2::GUS*, respectively, showed positive T‐DNA integration in the tea root genome. Blue staining in histochemical GUS assays further confirmed the presence in the transformed tea roots of *35S::GUS* and *35S::CsAFB2::GUS*, but not in the WT control (Figure 5a–c). We confirmed the overexpression of *CsAFB2* in the root samples by semiquantitative RT‐PCR analysis (Figure 5d). In the leaves of transformed tea lines, we also observed a significant up‐regulation of *CsAFB2* expression (Figure 5e). To further examine the function of *CsAFB2*, the leaves of non‐transformed control and transformed tea lines were inoculated with *C. gloeosporioides* to investigate its pathogenicity and the expression of *PR* genes. These tests showed that the leaves of control plants displayed the characteristic bigger brown lesions around wounded areas at 5 dpi. In contrast, transformed tea lines exhibited smaller lesions, suggesting a potential resistance response to *C. gloeosporioides* (Figure 5f). *PR1* was up‐regulated in *35S::CsAFB2::GUS* transgenic lines at 24 and 72 h after *C. gloeosporioides* infection compared to the WT plant (Figure 5g). In addition, the transcript levels of disease resistance‐related genes encoding phenylalanine ammonia‐lyase (*CsPAL*) and thaumatin‐like protein (*CsTLP*) were significantly induced in the leaves of transformed tea lines at 72 h post‐inoculation (hpi) with *C. gloeosporioides* (Figure 5h,i). These findings suggested that *CsAFB2* may play a role in plant resistance against *C. gloeosporioides*.

### 2.5 Overexpression of csn‐miR393a target mimic increases resistance of transgenic *N. benthamiana* to *C. gloeosporioides* infection

**FIGURE 6:**
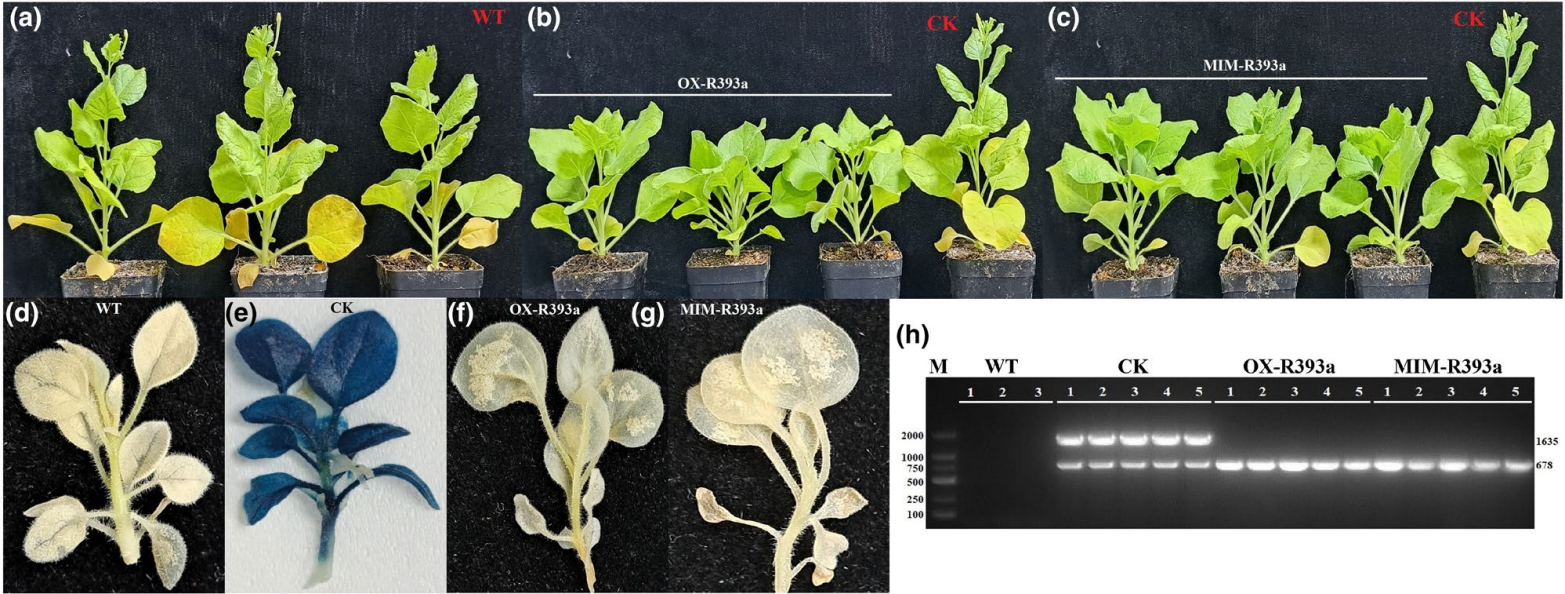
The developmental phenotypes of non‐transgenic (wild‐type, WT) and transgenic *Nicotiana benthamiana* at 8 weeks post‐germination (a–c) and selection of kanamycin‐resistant T_0_ transgenic *N. benthamiana* by β‐glucuronidase (GUS) expression (d–g). PCR analysis of marker genes, *gusA* (1635 bp) and *nptII* (678 bp) (h). CK, negative control.

**FIGURE 7:**
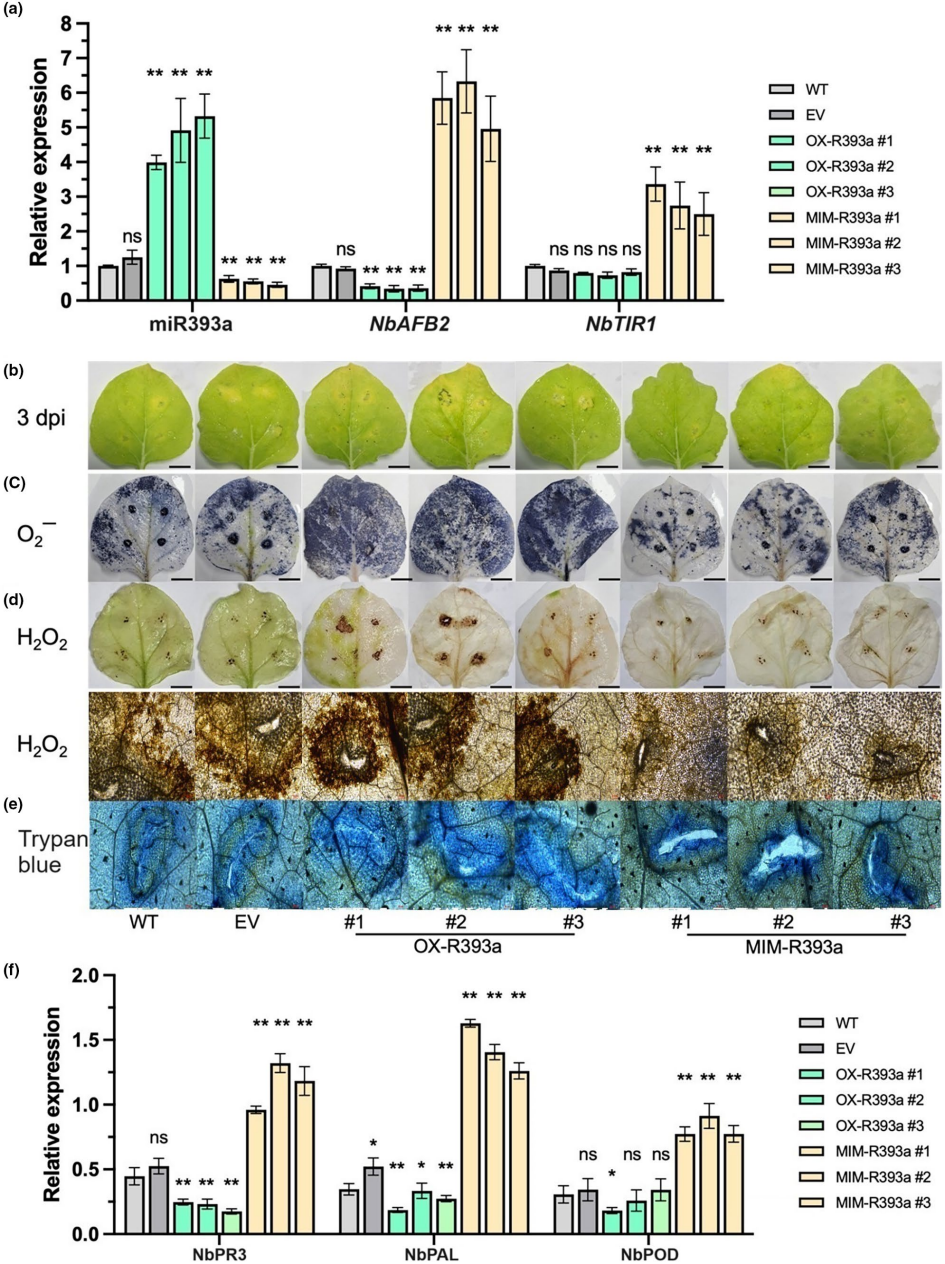
Overexpressing target mimic of csn‐miR393a leads to enhanced resistance to *Colletotrichum gloeosporioides* in *Nicotiana benthamiana*. (a) The expression of csn‐miR393a and targets of csn‐miR393a (*NbAFB2* and *NbTIR1*) in wild‐type (WT), empty vector control plant (EV) and transgenic lines overexpressing miRNAs (OX‐R393a) and its target mimic (MIM‐R393a). (b) Disease symptoms on the leaves of controls (wild‐type, WT; and empty vector, EV) and transgenic lines 3 days after inoculation with *C. gloeosporioides*. Analysis of $O_{2}^{-}$ with nitroblue tetrazolium staining (c), H_2_O_2_ with 3,3′‐diaminobenzidine staining (d) and cell death with trypan blue staining (e) in the controls and transgenic *N. benthamiana* leaves at 3 days after *C. gloeosporioides* inoculation. Bars (b–d) = 1 cm. (f) Expression analysis of defence‐related genes, *NbPR3*, *NbPAL* and *NbPOD* in transgenic lines overexpressing csn‐miR393a (OX‐R393a) and its target mimic (MIM‐R393a) in response to *C. gloeosporioides* infection. ns, not significant, *p < 0.05, **p < 0.01 (*t* test) compared to WT.

To investigate the regulatory role of csn‐miR393a in plant resistance to *C. gloeosporioides*, we generated transgenic *N. benthamiana* plants overexpressing csn‐miR393a (OX‐R393a) and its target mimic (MIM‐R393a) (Figure S3). We identified T_0_ transgenic lines by PCR analysis of *gusA* (1635 bp), *nptII* (678 bp) and *GUS* expression in the kanamycin‐resistant plants (Figure 6). Based on the accumulation of mature csn‐miR393a, we selected three T_1_ transgenic lines from each of the overexpressing constructs for further analysis. OX‐R393a and MIM‐R393a transgenic lines exhibited considerably increased and decreased csn‐miR393a abundance, respectively, compared to the WT plants (Figure 7a). Using psRNA Target server (https://www.zhaolab.org/psRNATarget/analysis), we identified that *NbAFB2* and *NbTIR1* could be targeted by csn‐miR393a in *N. benthamiana*. The mRNA levels of these targets showed that the *NbAFB2* expression was significantly decreased in the OX‐R393a transgenic lines in comparison with that in the WT plant and significantly increased in the MIM‐R393a transgenic lines. Although *NbTIR1* expression was increased in the MIM‐R393a transgenic plants, it was not changed in the OX‐R393a lines, indicating the inhibition of *NbAFB2* by csn‐miR393a in *N. benthamiana* (Figure 7a). To assess the role of csn‐miR393a in pathogen resistance, we inoculated the leaves of transgenic lines with 20 μL of *C. gloeosporioides* conidial suspension (2 × 10^5^ spores/mL) using the wound/drop inoculation methods. Three days after inoculation, necrotic symptoms with chlorotic haloes were more abundant in OX‐R393a transgenic lines than in the control lines, whereas MIM‐R393a transgenic lines inhibited the growth of *C. gloeosporioides* more effectively than the control plants by producing smaller chlorosis around wounded areas. The reduced chlorosis that developed on the leaves of the MIM‐R393a transgenic plants might be associated with enhanced disease resistance conferred by the target mimic of csn‐miR393a (Figure 7b).

To further investigate the levels of ROS in the leaves of transgenic lines and control plants, the leaves infected with *C. gloeosporioides* were incubated with NBT and DAB to detect $O_{2}^{-}$ and H_2_O_2_, respectively. At 3 dpi, the OX‐R393a transgenic lines produced obviously more ROS ($O_{2}^{-}$ and H_2_O_2_) than the control plants, whereas the levels of ROS were significantly decreased in the MIM‐R393a transgenic lines (Figure 7c–e). These results suggested that overexpression of the csn‐miR393a target mimic led to ROS homoeostasis being maintained and cell death being minimized in the transgenic lines after *C. gloeosporioides* infection. To understand why MIM‐R393a lines exhibited resistance to *C. gloeosporioides* infection, we further examined the relative expression levels of three defence‐related genes, *NbPR3* (chitinase), *NbPAL* and *NbPOD* (peroxidase), in the leaves of transgenic lines after *C. gloeosporioides* infection. The results showed that the expression levels of *NbPR3*, *NbPAL* and *NbPOD* were significantly induced in MIM‐R393a transgenic lines at 72 hpi with *C. gloeosporioides*, while in the OX‐R393a transgenic lines, *NbPR3* and *NbPAL* expression levels were decreased compared to the WT plants (Figure 7f). These results indicated that suppression of csn‐miR393a by its target mimic improves the resistance responses against *C. gloeosporioides* through promoting the expression of specific defence‐related genes in *N. benthamiana*.

## 3 DISCUSSION

miRNAs are crucial regulators of gene expression in regulating plant defence responses and innate immunity (Jeyaraj et al., 2020). During *C. gloeosporioides* infection, pathogen‐responsive miRNAs can fine‐tune the expression of target genes to improve the plant's disease resistance. In our previous study, *C. gloeosporioides*‐responsive miRNAs in tea plant were identified through high‐throughput sequencing (Jeyaraj et al., 2021). In this study, we established the pivotal role of csn‐miR393a in tea plants' resistance to *C. gloeosporioides* infection by suppressing *CsAFB2*. We used an antisense oligonucleotide (asODN) and short tandem target mimic (STTM) techniques to reveal the role of csn‐miR393a. Additionally, *A. rhizogenes*‐mediated tea root transformation was used to demonstrate the function of the csn‐miR393a target gene, *CsAFB2*. This transgenic system is challenging in tea plants due to the absence of stable transformation techniques developed for tea plants. Furthermore, we elucidated the role of csn‐miR393a in plant resistance against *C. gloeosporioides* infection by genetic transformation of *N. benthamiana* for an in‐depth study.

ROS, including H_2_O_2_ and $O_{2}^{-}$, are important signalling molecules that are essential for stress sensing and triggering plant defence mechanisms in plants, such as PR protein activation and hypersensitive response (HR)‐associated programmed cell death (PCD) to stop the spread of pathogens (Jain & Khurana, 2018; Mittler et al., 2022). Nevertheless, excessive ROS accumulation can result in oxidative stress, which is dangerous for plant biological systems. Previous studies demonstrated that elevated ROS levels cause cell death and tissue necrosis, which increases host susceptibility to necrotrophic pathogen infection by serving as a growth substrate for pathogen invasion (Tian et al., 2019; Xu et al., 2022). Higher ROS levels increased plant susceptibility to *Botrytis cinerea* infection (Asai & Yoshioka, 2009). Low concentrations of H_2_O_2_ have been shown to enhance resistance to *Colletotrichum camelliae* (Lv et al., 2023). Consistent with previous research, we observed reduced ROS accumulation and enhanced resistance against *C. gloeosporioides* in csn‐miR393a‐silenced tea leaves compared to the controls (Figure 4e).

Catechins, the main polyphenol compounds in tea plant, are naturally occurring antifungal substances that are crucial to a plant's defences against biotic stress (Jiang et al., 2015; Wang, Qian, et al., 2016). A previous study found that the content of total catechins was induced after *Colletotrichum fructicola* infection in tea plant, indicating that the EGCG and (+)‐C are crucial for tea plants' defence against anthracnose (Wang, Qian, et al., 2016). In vitro, it was demonstrated that EGCG, which comprises 76% of catechins, inhibits pathogens more potently than other catechin components (He et al., 2009; Ning et al., 2015). Our study showed that the content of EGCG was markedly increased in csn‐miR393a‐silenced tea leaves (Figure 4g), suggesting that the accumulation of catechins might be negatively regulated by csn‐miR393a in the response of tea plants against *C. gloeosporioides*.

Auxin, a phytohormone, is essential for the growth and development of plants as well as their defence against various pathogens (Zhang et al., 2019). *CsAFB2*, the target of csn‐miR393a, serves as an auxin receptor in the auxin signalling pathway. Auxin signals recognized by auxin receptors (*TIR1*/*AFB*) trigger transcriptional reprogramming in the auxin response pathway, which in turn activates *PR* genes and secondary metabolites to strengthen the plant's defences against pathogen infection (Jeyaraj et al., 2023; Jodder, 2020). PALs are essential proteins for the biosynthesis of phenolics and phytoalexins in response to insect and pathogen attacks, and TLPs, a class of PR proteins, have the ability to increase plant resistance against fungal diseases (Sun et al., 2020; Wang et al., 2019). In this study, these two defence‐related genes, *PAL* and *TLP*, were significantly induced in *CsAFB2*‐transformed tea lines at 72 hpi with *C. gloeosporioides* (Figure 5h,i), suggesting the auxin signalling pathway activated the defence‐related genes and enhanced the tolerance of tea plant to *C. gloeosporioides*.

Manipulating a single miRNA through transgenic technology has great potential to enhance the tolerance of plants to a variety of biotic stresses (Zhang & Unver, 2018). Because transformation techniques have not yet been developed specifically for tea plant, in this study, csn‐miR393a and its target mimic (STTM393) were overexpressed in *N. benthamiana* plants to further explore the functions in response to *C. gloeosporioides* stress. Three days after *C. gloeosporioides* inoculation, the OX‐R393a transgenic lines displayed more chlorotic symptoms, whereas the MIM‐R393a transgenic lines only had mild chlorosis compared to the control plants (Figure 7b). In addition, more cell death and higher ROS (H_2_O_2_ and $O_{2}^{-}$) levels were detected in the leaves of OX‐R393a transgenic lines compared to the control plants (Figure 7c–e). Previous studies demonstrated that the accumulation of ROS causes cell death and tissue necrosis, which promotes pathogen invasion by providing a growth substrate, thereby increasing host susceptibility during necrotrophic pathogen infection (Tian et al., 2019; Xu et al., 2022). Thus, the mild chlorosis with reduced cell death and ROS levels in MIM‐R393a transgenic lines suggest that overexpression of the csn‐miR393a target mimic leads to enhanced resistance against *C. gloeosporioides*.

The expression analysis of defence‐related genes was evaluated in all the transgenic lines in response to *C. gloeosporioides* infection. Compared to the controls, MIM‐R393a transgenic lines significantly induced *NbPR3*, *NbPAL* and *NbPOD* expression at 72 hpi with *C. gloeosporioides* (Figure 7f). Peroxidase (POD) is an important oxidoreductive enzyme in the biochemical defence mechanisms of plants and is also involved in plant metabolism after infection (Saravanan et al., 2004). The increased expression of *NbPOD* in MIM‐R393a transgenic lines might be associated with the lower ROS levels and cell death in these lines. The enzymes β‐1,3‐glucanase (*PR2*), chitinase (*PR3*) and phenylalanine ammonia‐lyase (*PAL*) are crucial for plant defence responses to pathogen infection: β‐1,3‐glucanase disintegrates cell walls of fungi; chitinase creates an effect against different fungal pathogens; and PAL produces antimicrobial phytoalexins (Appu et al., 2021). The highest expression of *NbPR3* and *NbPAL* in the MIM‐R393a transgenic lines suggests that overexpression of the csn‐miR393a target mimic enhanced the tolerance of *N. benthamiana* to *C. gloeosporioides*.

**FIGURE 8:**
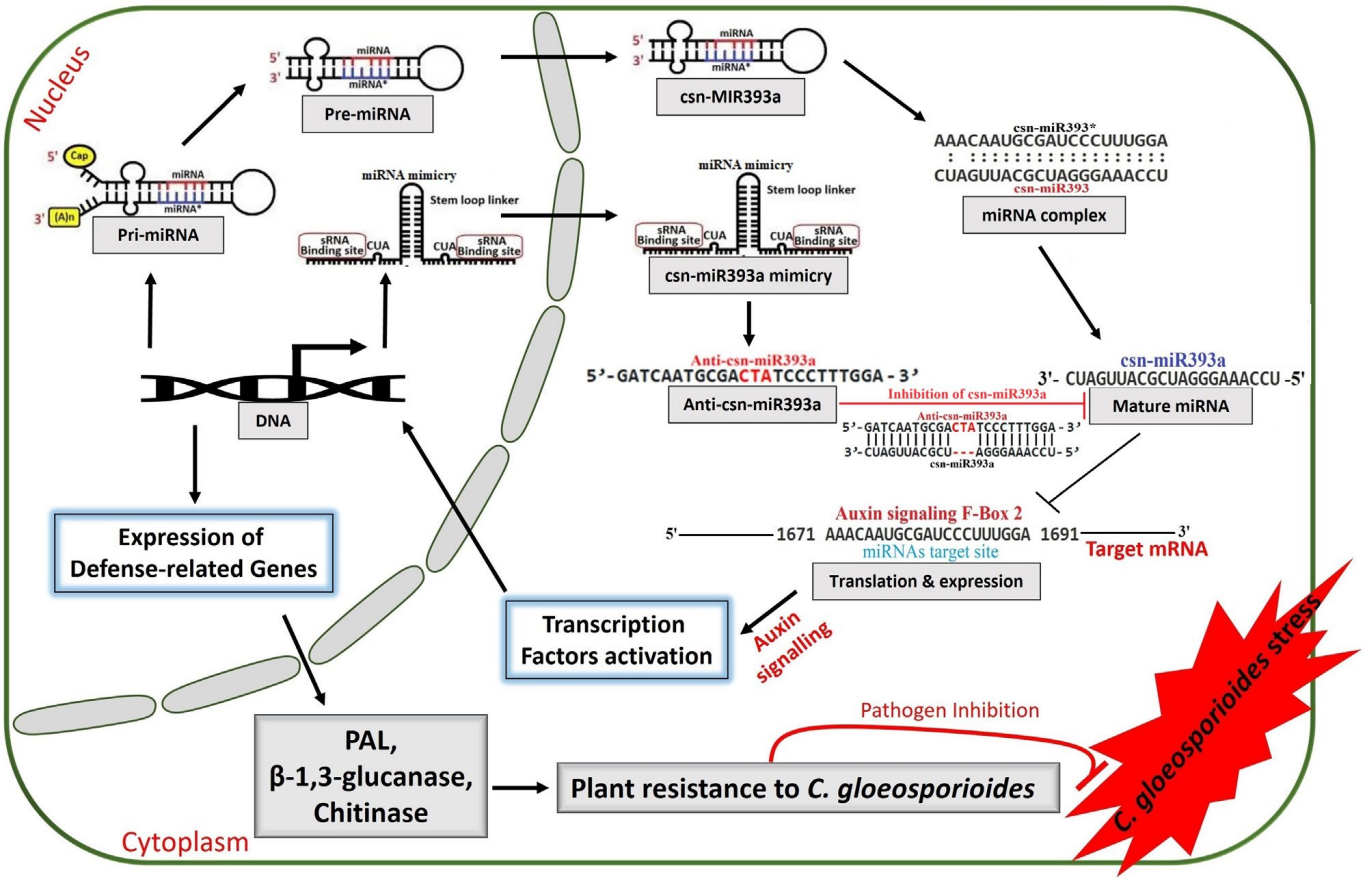
Working model for the role of csn‐miR393a‐CsAFB2 module in the plant defence response against *Colletotrichum gloeosporioides*. Elevated csn‐miR393a expression during *C. gloeosporioides* infection in Longjin43 (LJ43) directly reduces *CsAFB2* expression, which makes LJ43 more susceptible to infection. Overexpression of csn‐miR393a can be inhibited through the target mimicry mechanism, which progressively increases the translation and expression of *CsAFB2*, the target of csn‐miR393a; the enhanced expression of *CsAFB2* positively regulates the auxin signalling and activates the expression of defence‐related genes such as *PR*, *PAL* and *TLP*, thereby conferring tea plant resistance to *C. gloeosporioides* infection.

In summary, we demonstrated that csn‐miR393a negatively regulates the response of tea plant against *C. gloeosporioides* by affecting the accumulation of ROS, the expression of *PR* genes and the content of catechins. These results were consistent with the use of transgenic *N. benthamiana* plants overexpressing csn‐miR393a (OX‐R393a) and its target mimic (MIM‐R393a). In addition, overexpression of *CsAFB2* (auxin receptor), the target of csn‐miR393a, in the tea root system enhanced the tolerance of tea plants to *C. gloeosporioides*. Overall, we concluded that overexpression of a csn‐miR393a target mimic blocks the function of csn‐miR393a, thereby increasing the expression of *CsAFB2* (target of csn‐miR393a), which positively regulates the auxin signalling to enhance plant resistance (Figure 8). Therefore, overexpression of the csn‐miR393a‐specific target mimic or the csn‐miR393a target gene could be applied in anthracnose disease‐resistance breeding programmes for tea plants. The application of tea root transformation for *CsAFB2* overexpression will provide a foundation for future research to determine how increased auxin signalling regulates plant growth and development as well as resistance to abiotic stressors.

## 4 EXPERIMENTAL PROCEDURES

### 4.1 Plant growth conditions and pathogen inoculations

The tea plants used in this study were the susceptible cultivar Longjing43 (LJ43) and the resistant cultivar Zhongcha108 (ZC108). One‐year‐old LJ43 cuttings were used for asODN inhibition experiments and *A. rhizogenes*‐mediated root transformation. Wild‐type *N. benthamiana* plants were used to construct transgenic lines (OX‐R393a and MIM‐393a) and also used for *Agrobacterium*‐mediated transient expression. All the tea plants and *N. benthamiana* plants were maintained at 25°C and 75% relative humidity with 14/10 h of day/night photoperiod in an artificial climate chamber.

*Colletotrichum gloeosporioides* was isolated from diseased leaves of LJ43. The morphological and molecular characterizations of *C. gloeosporioides* were performed according to the method described by Jeyaraj et al. (2021). *C. gloeosporioides* was cultured on PDA and incubated at 28°C in darkness for 10 days to promote sporulation. Conidia were harvested and inoculum concentration was adjusted to 2 × 10^5^ spores/mL. Healthy tea leaves were inoculated with 20 μL of *C. gloeosporioides* conidial suspension using the wound/drop inoculation methods (Wang, Wang, et al., 2016). Leaves inoculated with sterile water were used as a control. After inoculation, each plant was enclosed in a plastic bag to maintain high relative humidity for conidial germination. Inoculation experiments were conducted independently and performed in three biological replicates, each of which consisted of five technical replicates (20 plants each in control and treatments). According to Jeyaraj et al. (2019), the healthy control and *C. gloeosporioides*‐inoculated leaves were harvested at 1, 4, 7 and 10 dpi. All samples were immediately frozen in liquid nitrogen and then stored at −80°C for further use.

### 4.2 Validation of gene expression and 5′ RLM‐RACE assay

Total RNA was extracted from the samples using RNeasy plant mini kit (Qiagen) according to the manufacturer's protocol. The quantity and purity of RNA samples were determined by using a NanoDrop2000 spectrophotometer (Thermo Fisher Scientific). Reverse transcription was done using PrimeScript RT Master Mix (Takara), with a specific stem–loop RT primer for csn‐miR393a (Varkonyi‐Gasic et al., 2007; Table S1) and oligo(dT) primer for target mRNA. RT‐qPCR was performed using SYBR Premix Ex Taq (Takara) with the appropriate primers (Table S1) as described previously (Jeyaraj et al., 2019). The small nuclear RNA (snRNA) *U6* and *actin* genes were used as the internal references for normalizing the expression of miRNAs and mRNAs, respectively. All RT‐qPCR analyses were performed in three biological replicates and the expression levels were calculated using the 2^−ΔΔ*C*t^ method (Livak & Schmittgen, 2001).

The cleavage site of csn‐miR393a in the target gene was verified by 5′ RLM‐RACE using the FirstChoice RLM‐RACE Kit (Invitrogen, Thermo Fisher Scientific) as described previously (Jeyaraj et al., 2019). The final RLM‐RACE products were gel‐purified, cloned into the pMD19 T‐vector (Takara), transformed into *Escherichia coli* DH5α competent cells (Vazyme) and sequenced. The sequencing results were analysed to map the cleavage sites.

### 4.3 Construction of recombinant vectors

The identified csn‐miR393a precursor sequences and the full‐length sequences of *CsAFB2* were used for this study (Jeyaraj et al., 2021). To construct recombinant miRNA vectors, DNA fragments containing the precursor of csn‐miR393a were amplified from LJ43 genomic DNA with primers miR393a‐BamHI‐F and miR393a‐SacI‐R (Table S1). The amplified fragments were digested and cloned into the BamHI‐SacI sites of the binary vector pBI121 to generate the recombinant overexpression construct *35S::MIR393a*. To construct the recombinant target gene vector, the open reading frame of *CsAFB2* was amplified without the stop codon using CsAFB2‐XbaI‐F and CsAFB2‐BamHI‐R primers (Table S1) and, then, cloned into the XbaI‐BamHI sites of the binary vector pBI121, resulting in overexpression construct *35S::CsAFB2*. For silencing csn‐miR393a, the artificial target mimic STTM393 was designed based on the previous method (Wu et al., 2013; Yang et al., 2021). The csn‐miR393a‐binding site motif of targets was used as a mimic target site, which contains a trinucleotide bulge (CTA) sequence in the middle of the miRNA binding site. A 96‐nucleotide construct of STTM393 containing two copies of the mimic target site (24 nucleotides) with a 48‐nucleotide linker was generated. All target mimic fragments were digested and cloned into the BamHI‐SacI sites of the binary vector pBI121, resulting in overexpression construct *35S:STTM393a*. The vector pBI121 contains the *GUS* reporter gene driven by the CaMV 35S promoter, which was used as the control. The constructed plasmids were used for leaf co‐transformation assays and genetic transformation.

### 4.4 Subcellular localization, co‐transformation and GUS assay

To determine the subcellular localization of the CsAFB2‐GFP fusion protein, the open reading frame of *CsAFB2* was amplified and cloned after the 35S promoter in the expression vector 35S‐pCAMBIA1300 to generate *35S::CsAFB2*‐*GFP* construct. The recombinant plasmids (*35S::CsAFB2*‐*GFP*) and the control plasmid (*35S‐GFP*) were introduced into *Agrobacterium tumefaciens* EHA105 competent cells to select a positive colony for infiltration of *N. benthamiana*. Green fluorescent protein (GFP) signals in the transiently infected leaves were observed using an upright confocal laser scanning microscope (LSM800).

For the co‐transformation assay, the individual expression constructs in the binary vector pBI121 were injected into the cells of *N. benthamiana* leaves using the *A. tumefaciens* GV3101‐mediated transfection system. GV3101‐pBI121 (control), GV3101‐pBI121‐pre‐miRNA (MIR393a), GV3101‐pBI121‐target mimic (STTM393a) and GV3101‐pBI121‐target (*CsAFB2*) were cultured to an optical density at 600 nm (OD_600_) of 0.8 prior to injection. After culturing, the bacteria were pelleted and resuspended in suspension buffer (10 mM MES‐KOH, pH 5.6, 10 mM MgCl_2_, 100 μM acetosyringone). The bacterial suspension was then adjusted to an OD_600_ = 0.5. Equal volumes of two or three bacterial suspensions were mixed, and the mixture was adjusted to OD_600_ = 1.0 for combined treatment. Finally, 1 mL suspension of each treatment was infiltrated into *N. benthamiana* leaves. After injection, seedlings were incubated in the dark at 25°C for 3 days (Feng et al., 2014; Wang et al., 2020). Histochemical staining and quantitative measurement of GUS activity in three independent biological replications were assayed as described by Jefferson et al. (1987).

### 4.5 Suppression of csn‐miR393a using asODNs

Oligodeoxynucleotide (ODN)‐based antisense repression of csn‐miR393a in tea plant leaves was conducted for the functional characterization of the candidate miRNA (csn‐miR393a) in response to *C. gloeosporioides* infection. The antisense oligonucleotides (asODNs) for csn‐miR393a were designed using Soligo software (Ding et al., 2004). To silence the selected miRNA expression, freshly detached healthy apical buds with first leaves from LJ43 were incubated in 1.5 mL Eppendorf tubes containing 1 mL of 100 μM asODN‐csn‐miR393a. Tea leaves incubated in water, random oligonucleotides or sense oligonucleotides (sODNs) were used as controls according to previously described methods (Wang et al., 2021; Yu et al., 2021). Then, the incubated tea leaves were inoculated with 20 μL of *C. gloeosporioides* conidial suspension (2 × 10^5^ spores/mL) by the wound/drop inoculation method (Wang, Wang, et al., 2016). At 48 hpi, the leaves were harvested for the detection of ROS, tea catechins and gene expression. All experiments were conducted in triplicate.

### 4.6 *Agrobacterium rhizogenes*‐mediated tea root transformation and functional analysis of *CsAFB2* under *C. gloeosporioides* stress

The binary vector pBI121‐gus and its recombinant vector, pBI121‐*CsAFB2*‐GUS, were introduced into *A. rhizogenes* (ATCC 15834) by electroporation (Nagel et al., 1990) and transformants were selected on TY solid medium (5 g tryptone, 3 g yeast extract, 10 mL of 1 M CaCl_2_ and 15 g agar in 1 L) supplemented with 50 mg/L kanamycin (Shanghai Weidi Biotechnology Co. Ltd). The cultures of *A. rhizogenes* (wild‐type) and *A. rhizogenes* containing pBI121‐GUS or pBI121‐*CsAFB2*‐GUS constructs were grown in TY liquid medium at 28°C overnight to mid‐log phase (OD_600_ = 0.5). These cultures were also streaked on TY solid medium and incubated for 4 days at 28°C to prepare *Agrobacterium* paste. *A. rhizogenes*‐mediated tea root transformation was performed as described previously with minor modifications (Alagarsamy et al., 2018; Mohanpuria et al., 2011). Briefly, the roots of 1‐year‐old LJ43 cuttings were excised and randomly punctured using a needle. Freshly wounded and unrooted tea cuttings were left in *Agrobacterium* culture at room temperature for 60 min for *Agrobacterium* infection. To enhance infection, the cut root tips were smeared with *Agrobacterium* paste and covered with cotton wool and planting sponge. The infected plants were transferred to the co‐cultivation liquid basal Murashige and Skoog (MS) medium (pH 5.6) supplemented with 200 μM acetosyringone and 2 mg/L 1‐naphthaleneacetic acid (NAA) for 2 days at room temperature in the dark. After co‐cultivation, the plants were potted in sand:soil mixture and maintained at 25°C with a 14/10 h of day/night photoperiod. To promote root formation, the plants were periodically watered with 1% liquid MS medium (pH 5.6) containing 2 mg/L NAA for 3 months.

To confirm the successful transformation, the non‐transformed control and transformed roots of tea were removed from the shoots of the cuttings. The collected roots were used for GUS histochemical staining assay according to Jefferson et al. (1987). PCR detection of the *GUS* gene in root DNA extracts was performed using gene‐specific primers (Table S1) to check the integration of transformed T‐DNA in tea roots. The expression level of *CsAFB2* was verified by semiquantitative RT‐PCR using gene‐specific primes (Table S1) according to the method described by Mohanpuria et al. (2011). PCR products were separated on 1% agarose gel and stained with ethidium bromide for visualization of the bands. Integrated band intensities were quantified in gel images using ImageJ (Schneider et al., 2012). The expression levels of csn‐miR393a and *CsAFB2* were verified in the leaves of control and transformed tea lines by RT‐qPCR analysis. To identify the role of *CsAFB2*, leaves of non‐transformed control and transformed tea lines were inoculated with *C. gloeosporioides* conidial suspension (2 × 10^5^ spores/mL). Infected leaves were harvested at 24 and 72 hpi and used for the analysis of *PR* gene expression to identify early responses of the plant to *C. gloeosporioides* infection.

### 4.7 Genetic transformation and analysis of transgenic *N. benthamiana* under *C. gloeosporioides* stress

The recombinant overexpressing constructs *35S::MIR393a* and *35S::STTM393a* were introduced into *A. tumefaciens* GV3101 and transformed into WT *N. benthamiana* plants using the leaf disc transformation method as described by Niedbała et al. (2021) with minor modification. The genomic DNA of the putatively transformed (kanamycin‐resistant) T_0_ lines was extracted, and the resulting DNA was used for PCR amplification of the selectable marker gene (*nptII*, *neomycin phosphotransferase*) and the reporter gene (*gusA*, *β‐glucuronidase*) with gene‐specific primers (Table S1). PCR products were separated on 1% agarose gel containing ethidium bromide, visualized and photographed under UV light. T_1_ generation seeds collected from PCR‐verified transformed T_0_ lines were surface sterilized and grown on MS medium supplemented with 200 mg/L kanamycin. The kanamycin‐resistant T_1_ seedlings were used for further analyses. WT and WT plants transformed with the empty vector pBI121 (EV) were used as a control. Two‐week‐old WT and transgenic seedlings with uniform growth were sown in soil and grown in an artificial climate chamber at 25°C with 14/10 h day/night photoperiod. The expression levels of csn‐miR393a and its target gene were verified in the leaves of control and transformed lines. For the disease‐resistance assays, 5‐week‐old WT and transgenic plants were inoculated with *C. gloeosporioides* conidial suspension (2 × 10^5^ spores/mL). Inoculated leaves were harvested after 3 days with three biological replicates for the analysis of defence‐related genes expression and histochemical staining assays.

### 4.8 Histochemical staining assay and determination of catechin content

In situ detection of hydrogen peroxide (H_2_O_2_) and superoxide radical ($O_{2}^{-}$) was performed according to an established protocol (Ramel et al., 2009). Briefly, tea leaves and *Nicotiana* leaves infected by *C. gloeosporioides* were immersed in freshly prepared 3,3′‐diaminobenzidine (DAB) solution (1 mg/mL, pH 3.8) and nitroblue tetrazolium (NBT) solution (2 mg/mL, pH 7.5), and incubated for 12 h in the dark at room temperature to measure H_2_O_2_ and $O_{2}^{-}$, respectively. To detect cell death, *C. gloeosporioides*‐treated leaves were immersed in 0.04% (wt/vol) lactophenol trypan blue solution overnight at 25°C (Li et al., 2016). After staining, the leaves were treated with 95% ethanol to remove chlorophyll and photographed. Microscopic images were captured using a Leica DM6B fluorescent microscope.

For catechin content determination, 0.2 g of powdered tea leaves was extracted with 10 mL of 70% (vol/vol) methanol at 70°C for 20 min. Then, catechins in the samples were determined according to the method described by Chen et al. (2015) using Acquity UPLC H‐Class Plus QSM (Milford). Authentic standards for epigallocatechin gallate (EGCG), epicatechin gallate (ECG), gallocatechin (GC), epigallocatechin (EGC), catechin (C), epicatechin (EC) and gallocatechin gallate (GCG) were purchased from Sigma‐Aldrich.

### 4.9 Bioinformatic and statistical analysis

Precursor sequences of csn‐miR393a and sequences of STTM393 target mimic were used to form folded hairpin structures using the Mfold web server (Zuker, 2003). The sequence logos of csn‐miR393a were generated using WebLogo (Crooks et al., 2004). The annotation of protein domains and physical properties of CsAFB2 were identified using PROSITE and ProtParam tools in the ExPASy server (https://www.expasy.org/). Multiple alignments of protein or nucleotide sequences were made with the program ClustalW using BioEdit (v. 7.2.5). Using MEGA (v. 11), the neighbour‐joining approach was used to construct the phylogenetic tree. The RT‐qPCR, tea catechins and GUS activity values obtained in this study are expressed as mean ± standard deviation values from three independent experiments. The statistical significances in Figure 1i were considered at the α = 0.05 level revealed by Duncan's multiple‐range test (DMRT) using DPS software (Jeyaraj et al., 2019).

## CONFLICT OF INTEREST STATEMENT

The authors declare they have no conflict of interest.

## Supporting information


**FIGURE S1.** The phylogenetic analysis and multiple sequence alignment of tea plant miR393a precursor (csn‐MIR393a) (a) and its target gene (*CsAFB2*) (b) with diverse plant species.

**FIGURE S2.** *Agrobacterium*‐mediated root transformation in tea cuttings using recombinant construct, pBI121‐*CsAFB2*‐GUS. (a) One‐year‐old LJ43 cuttings. (b) The roots of LJ43 were excised and randomly punctured using a needle. (c) Unrooted tea cuttings were infected with *Agrobacterium rhizogenes* (wild‐type) and *A. rhizogenes* harbouring pBI121‐GUS (control) and pBI121‐*CsAFB2*‐GUS constructs. The infected root tips were further smeared with *Agrobacterium* paste (d) and covered with cotton wool (e) and planting sponge (f). (g) *Agrobacterium*‐inoculated tea plants shifted to co‐cultivation on Murashige and Skoog medium. (h) Co‐cultivation under dark conditions for 2 days at room temperature. (i) After co‐cultivation, the infected cuttings transferred to growth chamber for effective transformation.

**FIGURE S3.** *Agrobacterium tumefaciens*‐mediated gene transformation of *Nicotiana benthamiana*. (a) Germinated seed on Murashige and Skoog basal medium. (b) Leaf explants on pre‐culture medium. (c) Leaf explants on selection medium after co‐cultivation with *Agrobacterium*. (d) Callus with regenerating shoot buds growing on selection medium. (e) Multiple shoots regenerating from the callus. (f) Growing shoots on callus. (g) Young plantlet with well‐developed roots obtained from the selection medium. (h) The acclimatization of the putative transgenic plant under greenhouse conditions. (i) Mature transgenic plants bearing flowers.

**TABLE S1.** All primers used in this study.

## Data Availability

All data supporting the findings of the current study are available within figures and supporting information.
