# Supplementary figures and images for: The regulation of auxin receptor gene CsAFB2 by csn‐miR393a confers resistance against Colletotrichum gloeosporioides in tea plants
Source: Mol Plant Pathol. 2025 Mar 28;26(4):e13499. doi: 10.1111/mpp.13499 (PMC11950636; doi:10.1111/mpp.13499)

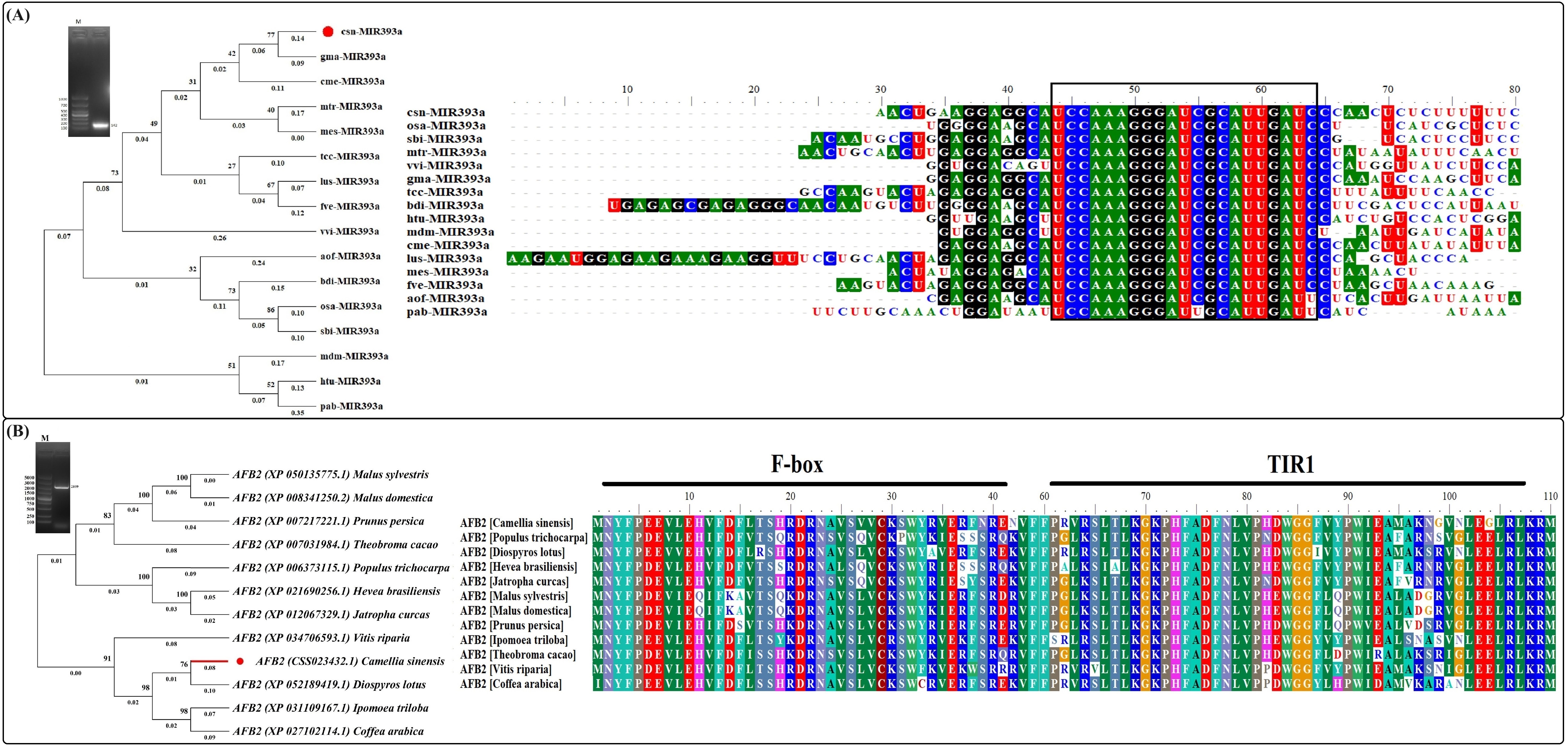

Supplement: Supplementary file 1 — FIGURE S1. The phylogenetic analysis and multiple sequence alignment of tea plant miR393a precursor (csn‐MIR393a) (a) and its target gene (CsAFB2) (b) with diverse plant species. [file MPP-26-e13499-s001.jpg]

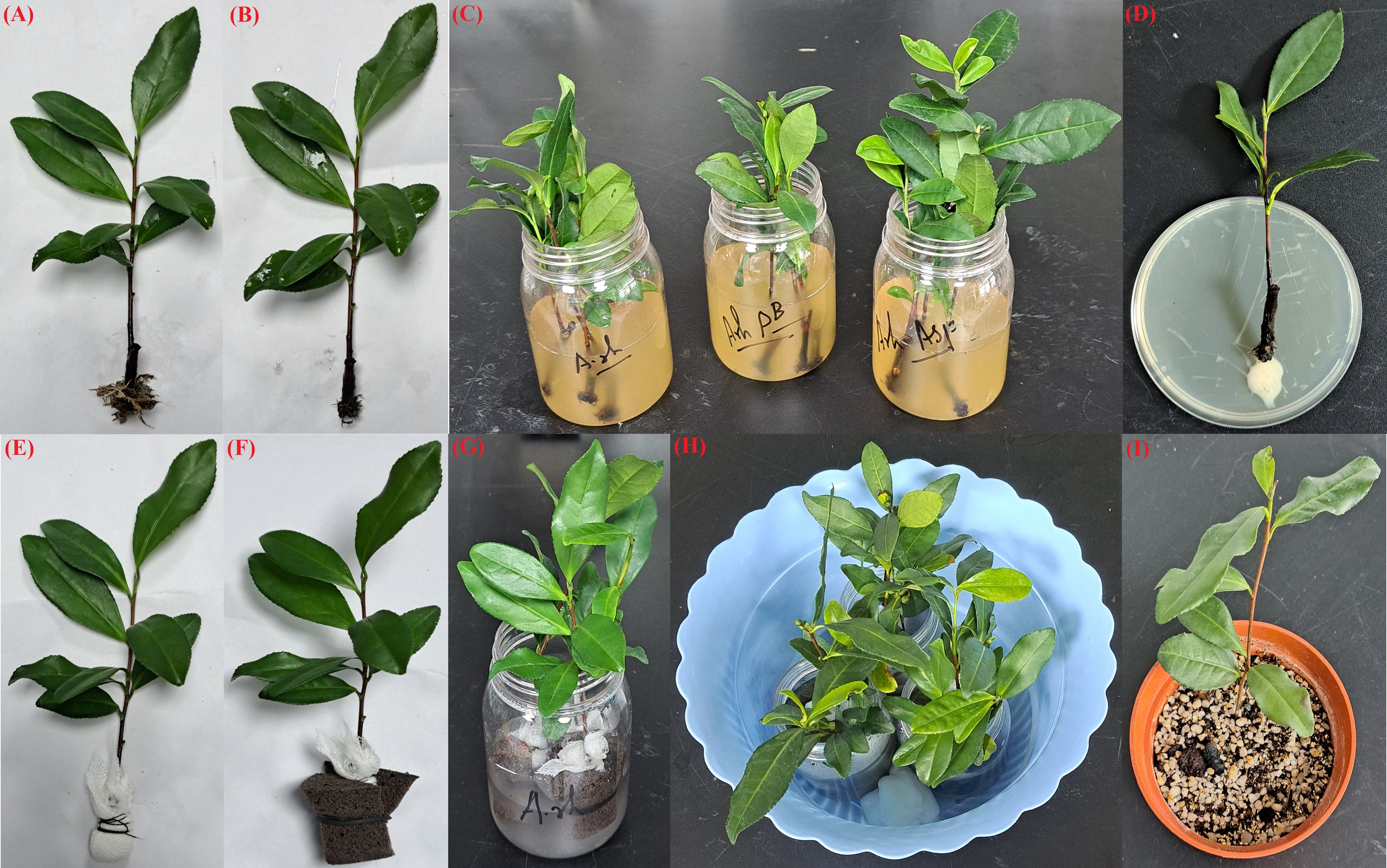

Supplement: Supplementary file 2 — FIGURE S2. Agrobacterium‐mediated root transformation in tea cuttings using recombinant construct, pBI121‐CsAFB2‐GUS. (a) One‐year‐old LJ43 cuttings. (b) The roots of LJ43 were excised and randomly punctured using a needle. (c) Unrooted tea cuttings were infected with Agrobacterium rhizogenes (wild‐type) and A. rhizogenes harbouring pBI121‐GUS (control) and pBI121‐CsAFB2‐GUS constructs. The infected root tips were further smeared with Agrobacterium paste (d) and covered with cotton wool (e) and planting sponge (f). (g) Agrobacterium‐inoculated tea plants shifted to co‐cultivation on Murashige and Skoog medium. (h) Co‐cultivation under dark conditions for 2 days at room temperature. (i) After co‐cultivation, the infected cuttings transferred to growth chamber for effective transformation. [file MPP-26-e13499-s002.jpg]

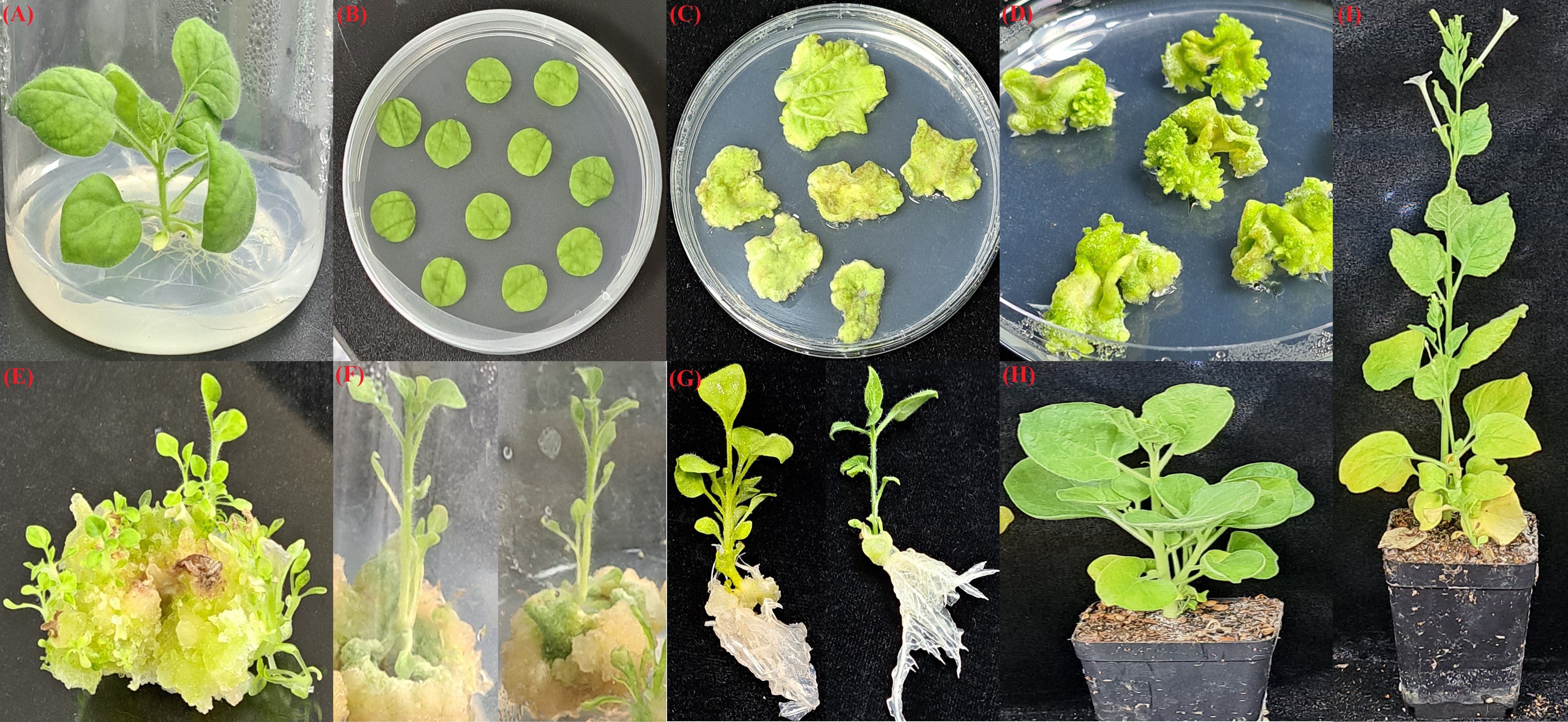

Supplement: Supplementary file 3 — FIGURE S3. Agrobacterium tumefaciens‐mediated gene transformation of Nicotiana benthamiana. (a) Germinated seed on Murashige and Skoog basal medium. (b) Leaf explants on pre‐culture medium. (c) Leaf explants on selection medium after co‐cultivation with Agrobacterium. (d) Callus with regenerating shoot buds growing on selection medium. (e) Multiple shoots regenerating from the callus. (f) Growing shoots on callus. (g) Young plantlet with well‐developed roots obtained from the selection medium. (h) The acclimatization of the putative transgenic plant under greenhouse conditions. (i) Mature transgenic plants bearing flowers. [file MPP-26-e13499-s004.jpg]
